# Structures of Microbial Communities in Alpine Soils: Seasonal and Elevational Effects

**DOI:** 10.3389/fmicb.2015.01330

**Published:** 2015-11-26

**Authors:** Anna Lazzaro, Daniela Hilfiker, Josef Zeyer

**Affiliations:** Environmental Microbiology, Institute of Biogeochemistry and Pollutant Dynamics, ETH ZurichZurich, Switzerland

**Keywords:** alpine habitats, seasonal variation, bacterial community composition, fungal communities, seasonal snowpack

## Abstract

Microbial communities in alpine environments are exposed to several environmental factors related to elevation and local site conditions and to extreme seasonal variations. However, little is known on the combined impact of such factors on microbial community structure. We assessed the effects of seasonal variations on soil fungal and bacterial communities along an elevational gradient (from alpine meadows to a glacier forefield, 1930–2519 m a.s.l.) over 14 months. Samples were taken during all four seasons, even under the winter snowpack and at snowmelt. Microbial community structures and abundances were investigated using Terminal Restriction Fragment Length Polymorphism (T-RFLP) and quantitative PCR (qPCR) of the 16S and 18S rRNA genes. Illumina sequencing was performed to identify key bacterial groups in selected samples. We found that the soil properties varied significantly with the seasons and along the elevational gradient. For example, concentrations of soluble nutrients (e.g., ${\text{NH}}_{4}^{+}\text{-N}$, ${\text{SO}}_{4}^{2-}\text{-S}$, ${\text{PO}}_{4}^{3-}\text{-P}$) significantly increased in October but decreased drastically under the winter snowpack. At all times, the alpine meadows showed higher soluble nutrient concentrations than the glacier forefield. Microbial community structures at the different sites were strongly affected by seasonal variations. Under winter snowpack, bacterial communities were dominated by ubiquitous groups (i.e., beta-Proteobacteria, which made up to 25.7% of the total reads in the glacier forefield). In the snow-free seasons, other groups (i.e., Cyanobacteria) became more abundant (from 1% under winter snow in the glacier forefield samples to 8.1% in summer). In summary, elevation had a significant effect on soil properties, whereas season influenced soil properties as well as microbial community structure. Vegetation had a minor impact on microbial communities. At every elevation analyzed, bacterial, and fungal community structures exhibited a pronounced annual cycle.

## Introduction

The high adaptive capacity and physiological flexibility of microorganisms allows them to colonize a huge variety of ecosystems. In soils, microbial communities react to environmental fluctuations by adjusting their functional and species composition to climatic and nutritional conditions (Grayston et al., 2001; Wallenstein and Hall, 2012). Seasons cause profound changes in factors such as temperature, humidity, vegetation and nutrient concentrations, which are crucial for microbial survival. Thus, in environments where such fluctuations are quite pronounced, the microbial communities are expected to show similarly pronounced changes (Monson et al., 2006; McMahon et al., 2011).

Alpine ecosystems experience a striking range of seasonal variations (Ernakovich et al., 2014). In these habitats, elevational gradients, seasonal snow cycles, and varied precipitation regimes affect plant species compositions and soil properties (Cebon et al., 1999), which influence the composition of the resulting microbial communities. Alpine ecosystems are therefore ideal platforms for exploring how microbial communities respond to environmental changes. Such studies also provide insights into how future changes in climate, which are expected to strongly influence alpine plant and microbial communities, will affect ecosystem functioning (Djukic et al., 2010).

Few studies have focussed on the dynamic changes of microbial communities in relation to meteorological factors and seasonal cycles. The extensive work of Lipson et al. (1999), and Lipson et al. (2002) described the general dynamics of the responses of microbial communities to seasonal changes, highlighting the interplay and community shifts of bacteria and fungi during snow-covered seasons.

In subnival alpine regions, soil is seasonally covered by snow, usually from November to May. Three major phases can be distinguished in the seasonal snowpack: (i) snow accumulation; (ii) steady-state; and (iii) snowmelt. Due to the dynamic properties of snow, each of the three phases may affect the covered soil in different ways in terms of thermal insulation, UV protection, light penetration, water availability, and nutrient input (Edwards et al., 2007; Libois et al., 2013; Lazzaro et al., 2015). During the snow-covered seasons, the physico-chemical properties of snow-covered soil vary (Buckeridge and Grogan, 2008, 2010) and affect the soil microbial communities. Indeed, past work on soil microbiology highlighted the existence of cold-adapted microbial life below the winter snowpack (Schmidt and Lipson, 2004; Buckeridge and Grogan, 2008). At spring snowmelt, meltwater flushing down the snowpack causes flushes of nutrients into the soils, affecting soil microbial biomass (Schmidt and Lipson, 2004; Edwards et al., 2007; Zinger et al., 2011).

In the context of global climatic change, moreover, alpine environments will experience shorter winter seasons, altered precipitation, and snowmelt regimes and thinner snowpack (Keller et al., 2005; Gobiet et al., 2014). From the viewpoint of soil microbiology it is essential to understand the impact of snowpack on bacterial and fungal communities. However, a comprehensive overview of temporal changes of snowpack properties combined with soil and microbiological analyses is lacking. Moreover, sampling at high elevation locations is typically performed in spring at the onset of snowmelt and very seldom in winter. In addition, most studies focus on high arctic soils and tundra (Buckeridge and Grogan, 2008, 2010), so little is known about the effect of seasonal snowpack on spatially complex alpine ecosystems (Lipson et al., 2000; Zinger et al., 2011).

In this study, we characterized bacterial and fungal communities at four different alpine sites representing an elevational gradient. The gradient ranged from an alpine pasture to high-elevation glacier forefield. Samples were taken over two consecutive years in summer and autumn as well as in winter from under the snowpack and in spring at snowmelt. We hypothesized that in alpine environments, (i) physico-chemical soil characteristics change with elevation and seasons, (ii). Structures of microbial communities change with seasons, and (iii) seasonal effects are more pronounced thanelevational effects (i.e., soil properties, vegetation). We combined the physico-chemical characterization of the soils with Terminal Restriction Fragment Length Polymorphism (T-RFLP) profiling of the 16S and 18S rRNA genes. Identification of bacterial populations responding to seasonal changes was performed through Illumina sequencing of the 16S rRNA gene.

## Materials and methods

### Sampling sites

We selected four sites [Börtli (B), Tätsch (T), Älpetli (A), Forefield (F)] at different elevations in the catchment of the Tiefen glacier, Canton Uri, Switzerland (Table 1, Lazzaro et al., 2015). The soils at B, T, and A are Podsols, whereas the soil at F is a shallow and gravelly Leptosol (Mausburger and Alewell, 2008). Each site is characterized by different grazing and vegetation patterns. T and B are grazed by cows between July and August, whereas A is a groundwater protection zone and no grazing is allowed. At F, due to its scarce vegetation, almost no grazing occurs at all, due to the scarce vegetation. Typical alpine grassland species are found at B and A (Table 1). At T only one dominant plant, *Alchemilla vulgaris* agg., is present. The glacier forefield site F, close to the glacier mouth, shows a pioneer plant vegetation typical for the location (Burga, 1999). In July 2014, plants were collected from a 1 m × 1 m area at each site, identified and oven-dried to estimate biomass (g m^−2^, Table 1) Plant biomass decreased with elevation from an average range of 163–804 g m^−2^ at T and A, to 3.5 g m^−2^ at F^.^. At B, plant biomass (131 g m^−2^) was probably underestimated due to grazing.

**Table 1:** **Location, elevation, and sampling dates of the four sites of this study**.

**Site**	**Abbreviation**	**WGS coordinates**		**Elevation m a.s.l**.	**Dominant plant species**	**Plant biomass (g dry plant m^−2^)**
		**N−**	**E−**			
Forefield	F	46.6094	8.4523	2519	*Salix herbacea* *Sagina saginoides* *Gaium megalospermum*	3.5
Älpetli	A	46.6003	8.4598	2269	*Crepis aurea* *Leantodon helveticus* *Nardus stricta* *Trichophorum caespitosum*	162.9 ± 65.1
Tätsch	T	46.5942	8.4712	2244	*Alchemilla vulgaris agg*	804.1 ± 504.6
Börtli	B	46.5888	8.4838	1930	*Alchemilla vulgaris agg* *Poa alpina* *Trifolium repens* *Trifolium nivale* *Ranunculs villarsii* *Ligusticum sp.* *Gentianella cg. germanica*	130.9

*Shading indicates snow-covered soils*.

**Figure 1:**
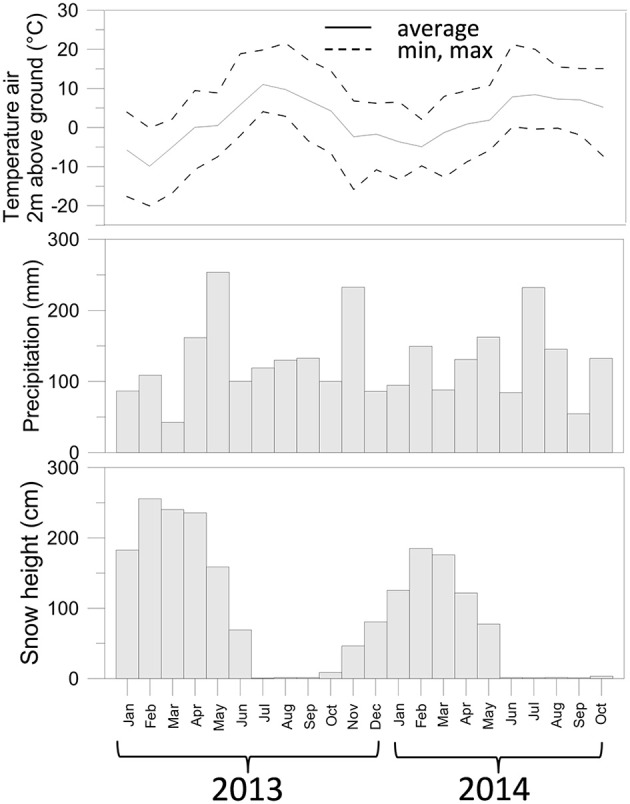
**Air temperature, precipitation, and snow depth measured for the sampling period of this study**. Data collected from the IDAWEB database of the Swiss Meteorological Institute (https://gate.meteoswiss.ch/idaweb/).

Meteorological data were obtained from the IDAWEB database of the Swiss Meteorological Institute (https://gate.meteoswiss.ch/idaweb/). Precipitation and temperature (Figure 1) were estimated by averaging data collected at the weather stations located at Grimsel Hospiz (WMO 06744, 1980 m a.s.l., 10 km SW from the catchment) and Gütsch ob Andermatt (WMO 06750, 2283 m a.s.l., 12.2 km NE of the catchment).

Total precipitation in the years 2013–2014 was overall similar (approx. 1600 mm), but was not distributed evenly over the months. In particular, May 2013 received more rainfall than May 2014 (Figure S1), while July 2013 received by far less than July 2014. In October 2012, moreover, temperatures dropped < 0°C (average −5.7°C over five consecutive days between Oct 27-Oct 31 2012, Figure S1), and temporary snowfall episodes deposited only a shallow layer of snow (average 9 cm), which could have caused freezing of the soil. In contrast, in Autumn 2013, the first 5 consecutive days with negative air temperatures (average −5.4°C) were from Nov. 19 till Nov. 23. At that time the average snow height was 73 cm and therefore sufficient to insulate the covered soil.

### Sampling conditions and procedure

At each location, we sampled five replicates along a 3 m transect at six time points from June 2013 to July 2014 (Table S1). Our sampling time points included snow-free summer and autumn, and snow-covered winter and early spring (snowmelt). In the snow-free seasons we ensured that sampling did not take place right after a meteorological extreme. On the sampling dates 23 August 2013, 25 October 2013, 27 July 2014, air temperatures were 10.9, 6, and 7.45°C, respectively (average elevation of 2131 m a.s.l.). The 5-day average temperature before each sampling date was 9.55, 5.81, and 8.72°C, respectively. The sampling took place on sunny or partly cloudy days. The average precipitation in the 5 days prior to sampling was < 5 mm/day. Such values maybe considered negligible, if compared to the 100 mm rain/day that are typically registered in alpine regions. During the snow-covered months, we exposed the soil by manually removing the snowpack manually with shovels and sampling the soil by using a previously sterilized spatula. At the vegetated sites, the samples were taken by removing the plants and surface soil and sampling the first 5 cm of soil. Each sample was stored in 50 ml Falcon tubes and transported to the laboratory under cold conditions. In the laboratory, aliquots were prepared for DNA-based analyses (stored at −20°C) and for physico-chemical characterizations of the soils (stored at 4°C until analysis, which was performed within 1 week of sampling). Replicate samples from each location were analyzed individually.

### Physico-chemical characteristics

Soil water content was estimated by allowing the samples to dry overnight at 80°C and then taking the difference between the sampled weight and the dry weight. A small number of soil samples taken from under the snow were accidentally mixed with the overlaying snow; these were not included in the water content calculations.

As the soils from B, T, and A contained tightly bound plant and root aggregates, we manually removed visible plant material with the aid of sterilized tweezers before passing the samples through a 6 mm sieve. Although the samples from F contained little or no plant material, they were also sieved.

To measure pH and soluble ions, extracts were prepared by combining 1 g of fresh soil with 5 ml of 0.01 M CaCl_2_(for pH and anions) or 2 M KCl (for cations) solutions, shaking them for 1 h and then briefly centrifuging (5 min at 5000 g) them to allow the soil particles to settle to the bottom of the tubes.

Soil pH was measured in the supernatant of the CaCl_2_ extracts with a Metrohm pH meter (Metrohm, Zofingen, Switzerland). Anion (${\text{NO}}_{3}^{-}$, ${\text{SO}}_{4}^{2-}$) concentrations were measured with a DX-320 ion chromatograph (Dionex, Sunnyvale, CA). In the supernatant of the KCl extracts, ${\text{NH}}_{4}^{+}$ concentrations were measured colorimetrically according to Mulvaney (1996). To measure dissolved organic carbon (DOC), water extracts were prepared by adding 1 g of soil to 10 ml of dd water and shaking overnight. The extract was syringe-filtered through a 0.45 μm PES filter (VWR), acidified with 40 μL of 37% HCl and measured with a Shimadzu TOC analyzer (Shimadzu GmbH, Reinach, Switzerland). The same TOC analyzer was used to measure Total carbon (TC) in selected dried and milled soil samples (each 100 mg).

### DNA extraction and T-RFLP

DNA was extracted from 0.5 g samples of soil using a MoBio Ultraclean® Soil DNA extraction kit (Mobio, Carlsbad, CA). The extracted DNA was quantified with a Nanodrop UV-Vis spectrophotometer (NanoDrop products, Wilmington, DE).

Aliquots of the extracted DNA (2–5 ng) were PCR-amplified with primers targeting the bacterial (primer pair 27F-1406Rev; Winsley et al., 2012) 16S rRNA gene and the fungal (primer pair nu-SSU-0817-FR1; Borneman and Hartin, 2000; Chemidlin Prévost-Bouré et al., 2011, Table S2) 18S rRNA gene. All of the forward primers used were FAM-labeled at the 5′ end. PCR reactions included a master mix containing 1X DreamTaq PCR buffer, 0.5 μM of each primer, 0.2 mM dNTPs, 2U DreamTaq polymerase and the template DNA, all in a 25 μl end volume. All reagents were supplied by Fermentas (Wohlen, Switzerland). For the bacterial 16S rRNA gene amplification, PCR consisted of a first step at 94°C for 2 min followed by 35 cycles of 94°C for 30 s, 56°C for 45 s, and 72°C for 1 min. The reaction was terminated by a final elongation step at 72°C for 4 min.

For the fungal 18S rRNA gene amplification, we applied a touchdown PCR protocol that included the same steps as before, but employed a −0.5°C decrease at each cycle from 56 to 50°C, and 12 cycles at 50°C. Through repeated testing of PCR conditions, we concluded that a 35-cycle PCR would provide us with consistent PCR products that could be efficiently analyzed by T-RFLP.

Positive PCR products were validated through agar gel electrophoresis and GelRed staining (Invitrogen, Life Technologies, Zug, Switzerland). Through preliminary comparisons with different restriction enzymes (MspI, HaeIII, and AluI), we found that the community structure analyses gave comparable results. We also found that AluI provided us with good community patterns (in terms of the number of OTUs detected) for both the bacterial 16S rRNA gene and the fungal 18S rRNA gene. This enzyme has been adopted before for both bacterial and fungal communities (Zumsteg et al., 2012). All the PCR products were therefore digested with equal volumes of the restriction enzyme AluI in 1% Y+ Tango buffer (Fermentas) and purified with Millipore Montage SEQ96 purification plates (Millipore, Billerica, MA) according to the manufacturer's instructions.

Lastly, 3 μL of digestions were added to 10 μL of HIDI Formamide (Applied Biosystems ABI, LifeTechnologies, Zug, Switzerland) and 0.1 μL of ROX 1000 standard (Bioventures Inc., Murfreesboro, TN), denatured for 2 min at 95°C and immediately placed on ice. Terminal restriction patterns were obtained by capillary electrophoresis on an ABI 3130XL sequencer (ABI) and analyzed with Genemapper software version 3.7 (ABI).

### qPCR

Quantitative PCR was performed on bacterial 16S rRNA and fungal 18S rRNA. For bacterial 16S rRNA genes, we used the primer pair 349F/806 Rev (Takai and Horikoshi, 2000, Table S2). For the fungal 18S rRNA gene we used the primer pair FR1/FF390 (Chemidlin Prévost-Bouré et al., 2011, Table S2).

The PCR assays were conducted in an ABI Prism 7000 sequence detection system (Applied Biosystems). Each reaction contained the following: 8.2 μl of dd water, 10 μl of KAPA SYBR® FAST qPCR Kits (Kapa Biosystems Inc. Wilmington, MA), 0.4 μl of each primer (10 μM) and 1 μl template DNA (0.5 ng μl^−1^), for a 20 μl end volume. PCR conditions applied a first 2 min step at 50°C; then 10 min at 95°C; followed by 35 cycles of 95°C for 15 s, 59°C for 1 min, 95°C for 15 s; and a final dissociation step of 95°C for 15 s, 60°C for 1 min, 95°C for 15 s, and 60°C for 15 s.

Each reaction was repeated in triplicates. Melting curve analysis of the PCR products was conducted to confirm specific amplification of the target gene.

For bacterial and fungal standards, we used dilution series of PCR products derived from DNA extracted from the appropriate positive control strain (*Methylococcus capsulatus* for bacteria, *Penicillium chrysogenium* for fungi, supplied by I. Brunner, WSL Birmensdorf) in a range between 4.5 e^4^ and 4.5 e^12^ gene copies μl^−1^ for bacteria and between 2.5 e^2^ and 2.5 e^8^ gene copies μl^−1^ for fungi. Target copy numbers for each reaction were calculated from the standard curves, assuming that the average molecular mass of a double-stranded DNA molecule is 660 g mol^−1^.

We are aware that 16S rRNA and 18S rRNA gene copy numbers per cell may vary in different species and reflect different growth conditions (Farrelly et al., 1995). This measurement, nethertheless, permitted us to estimate fluctuations in the genomic pool of the different samples.

### Illumina miseq sequencing of the 16S rRNA gene

16S rDNA libraries were constructed with the seasonal samples from sites A and F, which have the most contrasting conditions. For the analysis, the five replicates within each sample sets were pooled and quantified with Qubit® (Life Technologies, Zug, Switzerland). The V3–V4 regions of the 16S rRNA gene were PCR-amplified by using the universal forward primer Bakt_341F (Accession Number pB-03844) and the reverse primer Bakt_805R (pB-03845; Herlemann et al., 2011, Table S2). The PCR reaction mixtures consisted of 18 μl of 1X KAPA HIFI Hotstart Readymix, 0.3 mM of each primer, 5% of DMSO 100%, PCR-grade water and 2 μl (1–100 ng) of DNA template. PCR conditions applied a first denaturation step at 95°C for 5 min; 10 cycles of 98°C for 20 s, 60°C for 15 s, and 72°C for 15 min; and a final step at 72°C for 5 min.

Following purification with AMPure beads (Agilent Technologies Santa Clara, CA), according to the manufacturer's instructions, the PCR products were quantified with Qubit®. For indexing, each PCR product (0.1–10 ng) was mixed in 18 μl of a PCR master mix containing 1X KAPA Hi Fi Hot Start Ready mix, Nextera indexing primers (Illumina, San Diego, CA), 5% DMSO and PCR-grade water to 25 μl end volume. PCR included an initial step of 95°C for 3 min and eight cycles of 95°C for 30 s, 55°C for 30 s, and 72°C for 30 s. The reaction ended with a final step at 72°C for 5 min. The products were purified once more with the AMPure beads before performing fragment quality control on a bioanalyzer chip and quantifying the samples through qPCR. Finally, the sequencing library was prepared for analysis on an Illumina MiSeq sequencing platform at a final concentration of 4 nM. The reads were processed based on the pipeline designed at the GDC (ETH Zurich, Switzerland, Table S3).

The sequences were submitted to the European Nucleotide Archive (ENA) under accession number PRJEB9659 (Release date Dec 2015).

### Statistical analysis

Split-plot ANOVA was performed on the physico-chemical soil parameters, setting site and season as between-subjects and within-subjects factors, respectively. In addition, significances of the means of all measured parameters at each site were estimated separately with repeated measures ANOVA (rANOVA) by setting season as a within-subjects factor. All analyses were performed with Systat V.12.

Prior to the statistical analyses, the raw T-RFLP electropherograms were converted into relative abundance profiles by relating each fluorescence peak height to the total fluorescence detected in the profile. Seasonal microbial community dynamics were estimated on the basis of the normalized T-RFLP profiles. PcoA based on Bray-Curtis dissimilarity were performed with the vegan package R (Oksanen et al., 2005). In addition, we performed a time lag analysis based on Bray-Curtis distance matrices as described in Collins et al. (2000) and in Kampichler and van der Jeugd (2013). A Mantel test with Pearson's correlation coefficient was used to estimate the significance of the resulting slope.

## Results

### Physico-chemical characterization of the sampling sites

**Figure 2:**
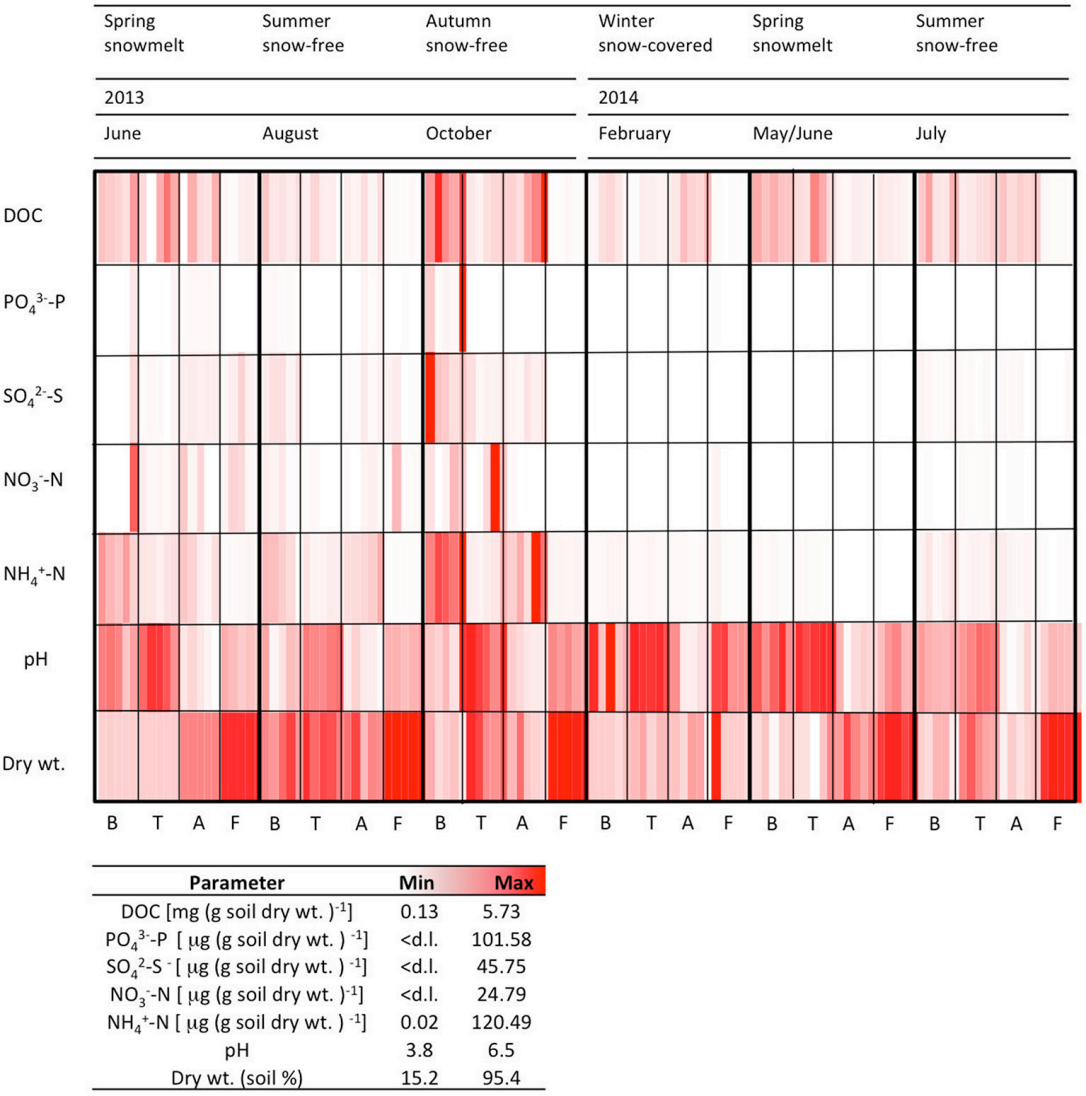
**Physico-chemical characteristics for five replicates measured at each time point at each site**. d.l. = detection limit (0.1 μg g soil dry wt^−1^).

The measured chemical parameters measured showed high significant variability between the sites (Figure 2, Table 2). TC content ranged from 5.7 to 27.5% at B, T and A, but it was always < 1% in the F samples (data not shown). DOC concentrations were higher at the vegetated sites B, T, and A than at the sparsely vegetated site F. Maximum DOC values (5.7 mg DOC g soil dry wt^−1^) were found in the samples from A. In the samples from F, concentrations were an order of magnitude lower (average 0.1 mg DOC g soil dry wt^−1^). ${\text{PO}}_{4}^{3-}\text{-P}$ was highly variable, with a peak of approximately 100 μg ${\text{PO}}_{4}^{3-}\text{-P}$ (g soil dry wt^−1^) at site B. ${\text{NO}}_{3}^{-}\text{-N}$ was present in similar concentration in all of the soils, ranging from below the detection limit to a range between 5.07 and 24.79 (T) μg ${\text{NO}}_{3}^{-}\text{N}$ (g soil dry wt^−1^). ${\text{NH}}_{4}^{+}$ concentrations were highest at B and A, where they reached values of about 120 μg ${\text{NH}}_{4}^{+}\text{-N}$ (g soil dry wt)^−1^. At all sites, soil pH was generally acidic (3.8–6.5), with the lowest values at A (Figure 2). Site F was always had the lowest water content (Figure 2).

**Table 2:** **Split-plot ANOVA (site and season as categorical variables) and rANOVA analyses of the physico-chemical characteristics and microbial parameters between site and seasons and within each site along the seasons**.

	**Split-plot ANOVA**			**rANOVA**			
	**Site**	**Season**	Site ^*^ Season	**B**	**T**	**A**	**F**
DOC	^***^	^**^	^***^	^***^	^*^	^*^	^***^
${\text{PO}}_{4}^{3-}$	^***^	n.s.	n.s.	n.a.	^***^	^**^	^***^
${\text{SO}}_{4}^{2-}$	^***^	n.s.	^***^	^***^	^***^	^***^	^***^
${\text{NO}}_{3}^{-}$	^***^	n.s.	^**^	^*^	^**^	n.s	n.s
${\text{NH}}_{4}^{+}$	^***^	n.s.	n.s.	^***^	^*^	^**^	^**^
pH	^***^	^***^	^***^	n.s.	^***^	n.s	^**^
Soildry weight	^***^	^***^	^***^	^***^	^***^	^***^	^***^

* p < 0.05;

** p < 0.01;

*** p < 0.001; n.s.,

*p > 0.5; n.a., not available*.

### Seasonal variations within each sampling site

The soil related parameters showed strong seasonal fluctuations (Figure 2). Striking increases in the concentrations of the measured soluble ions were found in autumn (October 2013) and spring (June 2013). Interestingly, increases in spring 2013 were not as pronounced in the spring of the following year (May/June 2014). TC varied seasonally, and was lowest in the February samples (approx. 11.6–13.8% in the B, T and A samples, and 0.02% at F). The highest pH was observed under the winter snowpack (B, T, and F).

The dry weight of the soils showed strong seasonal variation. At B, T and A, the soils had a higher water content under winter snow and at snowmelt, but they were drier in the summer months of 2013 (Figure 2). In July 2014, sampling was performed after a rain event, and this resulted in wetter samples.

### Bacterial and fungal community comparison between the different sampling sites

**Figure 3:**
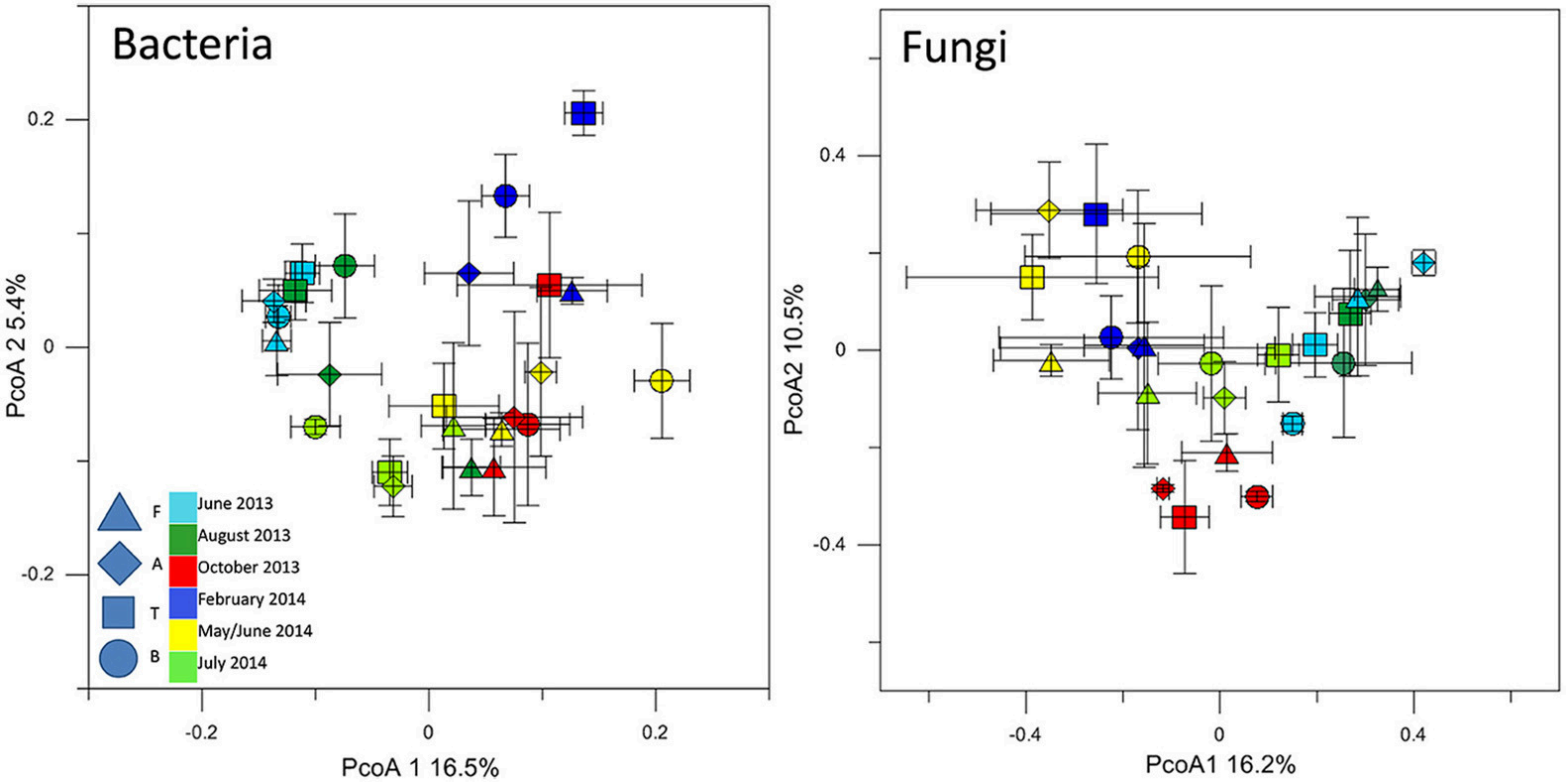
**PcoA plot with Bray-Curtis dissimilarity based on T-RFLP profiles of the bacterial 16S rRNA gene and of the fungal 18S rRNA gene**. Symbols indicate average PcoA coordinates, and bars indicate the distribution of each replicate.

The T-RFLP analysis of bacterial community structures at all sites and timepoints did not reveal great differences between the soils. Most of the samples clustered together (Figure 3) regardless of site, indicating the presence of the same dominant OTUs in all of the community profiles. A detailed inspection of the bacterial T-RFLP profiles showed that, within each season, all the samples shared generally 33–62% of the OTUs, except for February (8.9% average shared OTUs). The PcoA of the fungal T-RFLP profiles (Figure 3) showed a similar pattern. However, only a small percentage of the OTUs was shared by all four sites within each season (3–11.8%, data not shown). The shared OTUs belonged to the most dominant ones.

### Seasonal bacterial community composition within each site

**Figure 4:**
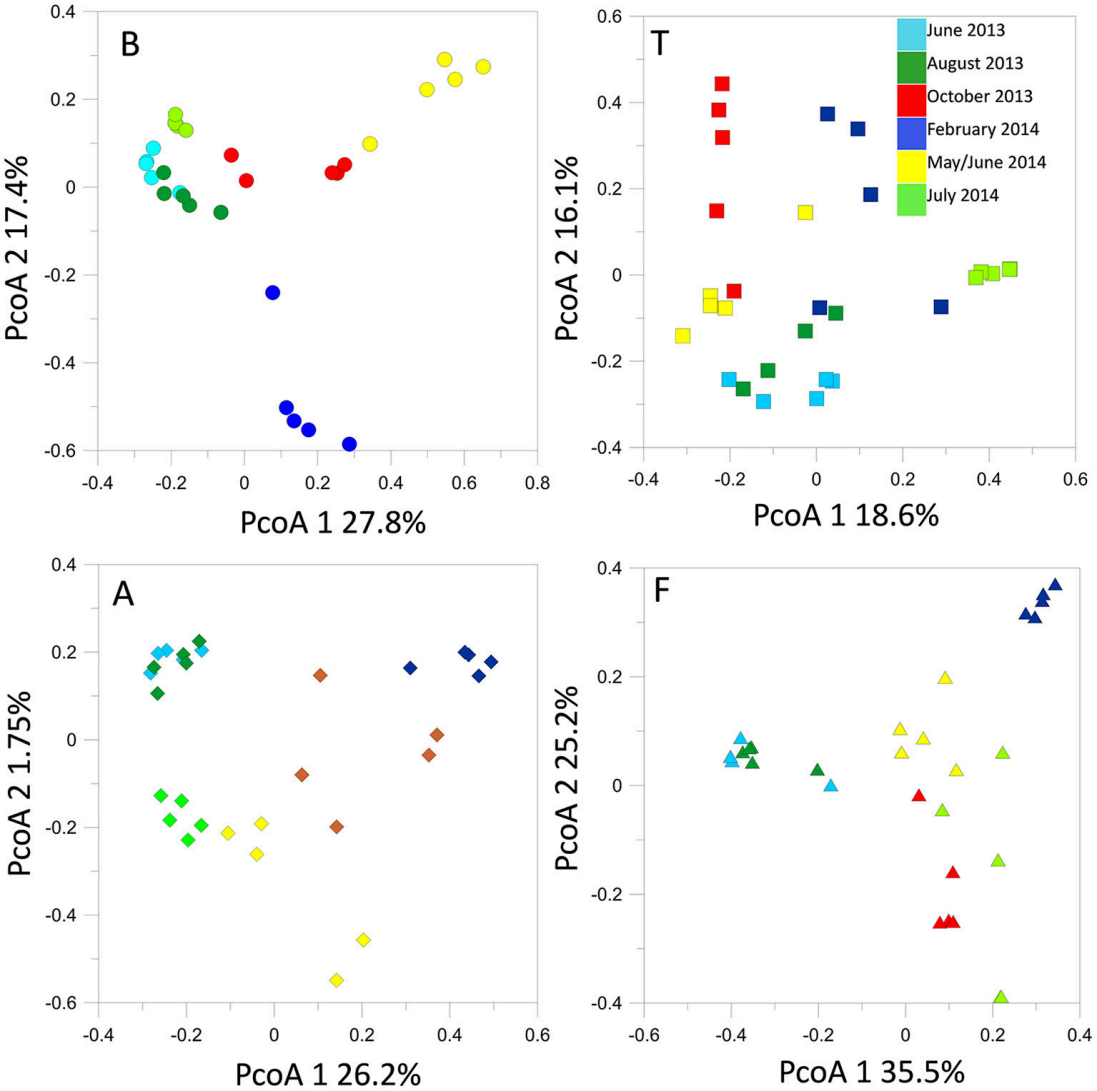
**Bacterial 16S rRNA gene T-RFLP profiles within each sampling site, represented by PcoA plots**.

T-RFLP profiling revealed seasonal fluctuations in OTU numbers and community shifts at the different sampling seasons. At the beginning of our sampling campaign (June 2013) the bacterial communities were quite similar between sites. However, pronounced shifts appeared in the T-RFLP profiles from October 2013 to June 2014 (Figure 4). In particular, OTU numbers decreased drastically in February 2014. Similar fluctuations were observed in the relative abundances of the dominant OTUs in the T-RFLP profiles for all sites and for all seasons (data not shown). Analyses of the seasonal shifts within each site indicated that the samples of July 2014 tended to return more similar to their previous year counterparts at all locations except for at F.

Time lag analysis based on Bray-Curtis dissimilarities evidenced significant regression lines, the direction and slope of which can be related to the dynamics of community structures (Kampichler and van der Jeugd, 2013). We observed significant (*p* < 0.01) convergence trends in the communities of B, T, and A to convergence. In contrast the bacterial communities at F tended to diverge (Figure S2).

**Figure 5:**
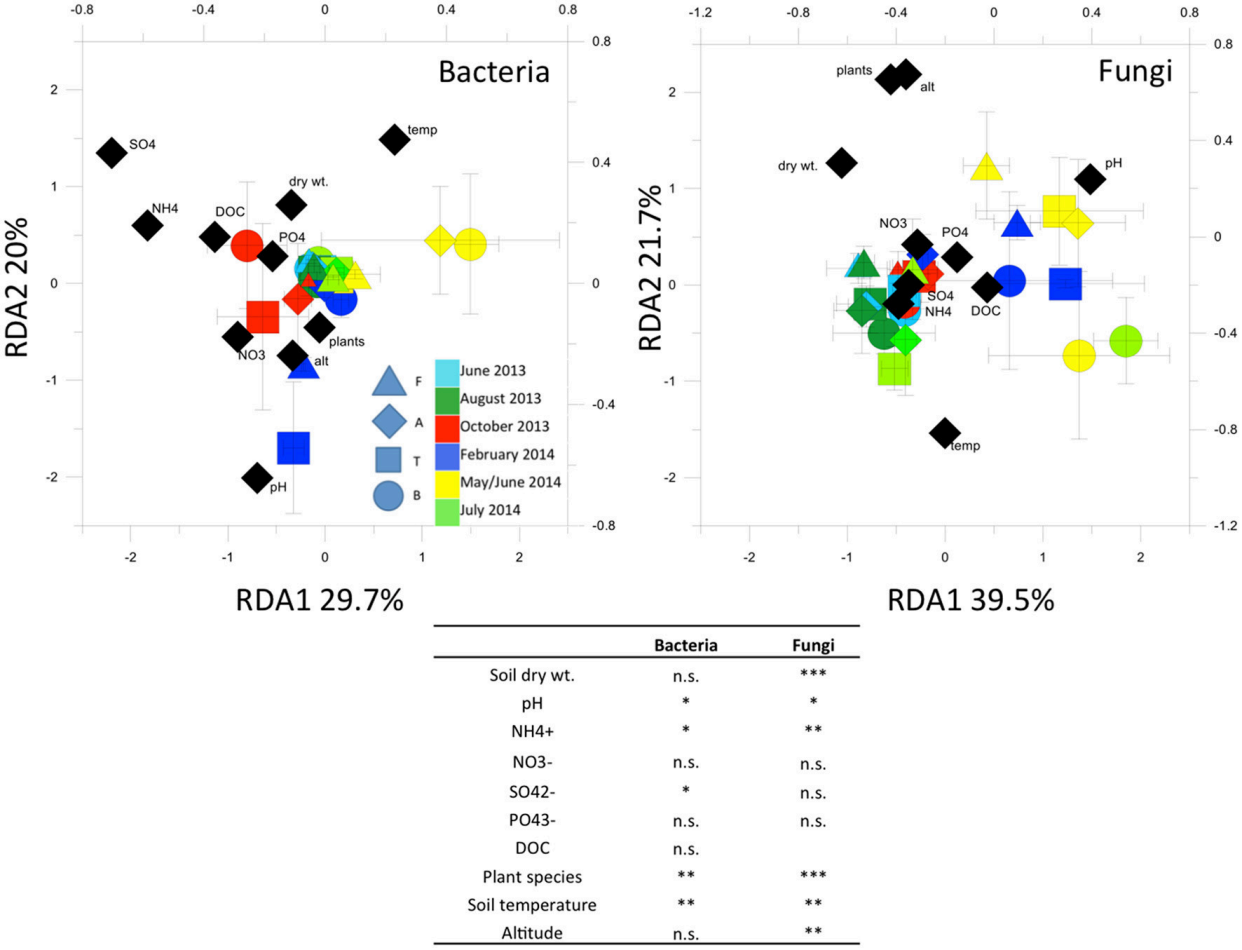
**Redundancy analysis (RDA) of bacterial and fungal 16S and 18S rRNA gene T-RFLP profiles**. In the table, Permutation test for RDA under reduced model (999 permutations)Asterisks indicate significant correlations at the 0.05 level. ^*^p < 0.05; ^**^p < 0.01; ^***^p < 0.001; n.s. = not significant (*p* > 0.05).

RDA analysis (Figure 5) highlighted important environmental factors related to the bacterial T-RFLP profiles. The environmental factors we included in the analysis explained 15% of variance in the T-RFLP profiles. Soil temperature, pH, ${\text{SO}}_{4}^{2-}$ and ${\text{NH}}_{4}^{+}$ concentrations were the most significant factors found.

### Seasonal fungal community composition

**Figure 6:**
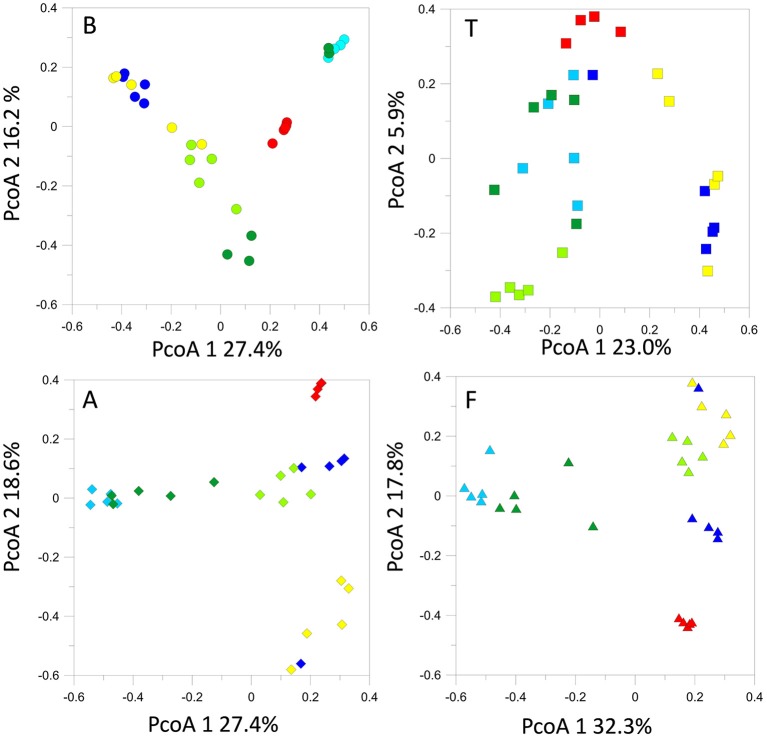
**Fungal 18S rRNA gene T-RFLP profiles within each sampling site, represented by PcoA plots**.

The structures of the fungal communities estimated with T-RFLP profiling of the 18S rRNA gene evidenced high seasonal dynamics at all locations. Generally, detected OTU numbers were low (< 70 OTUs). Maximum OTU levels were reached in October 2013 at sites B, A, and T. At B, A and F, we observed a seasonal cycle, in which the spring-summer 2014 fungal community tended to return to structures similar to those observed during the previous spring-summer (Figure 6). However, time lag analysis (Figure S2) showed that the communities were in a dynamic state and tended to diverge at all locations. All the samples displayed the same dominant OTUs, the relative abundances of which fluctuated in the same way with the seasonal course (data not shown).

RDA (Figure 5) showed that the factors we included in the analysis explained 16.6% of variance in the fungal T-RFLP profiles. Plant species, soil temperature, pH and ${\text{NH}}_{4}^{+}$ concentrations, as well as soil humidity, were most significantly related to the community profiles.

### Seasonal abundance of bacterial and fungal rRNA genes

Quantitative PCR showed highest bacterial 16S rRNA copy numbers at B and A, which had an average copy number in the range of 10^10^ and 10^11^ gene copies·g soil dry wt^−1^, respectively. The lowest numbers were found at F, where copy numbers fell to a range between 10^6^ and 10^10^ gene copies·g soil dry wt^−1^. At all locations, we observed strong seasonal fluctuations. For example, a decline in the copy numbers of the 16S rRNA gene was observed in all of the February 2014 samples (9.6 10^6^–9.3 10^8^ gene copies·g soil dry wt^−1^). In contrast, we observed the highest copy numbers in August 2013 (B, 5.3 10^11^, A, 6.0 10^11^ gene copies·g soil dry wt.^−1^).

Fungal 18S rRNA gene copy numbers (data not shown) were generally lower, in a range between 10^6^ and 10^10^ gene copies·g soil dry wt^−1^ in all the samples. Fungal gene copy numbers were the lowest at F, with an average copy number of approximately 10^7^ gene copies·g soil dry wt^−1^. Copy numbers fluctuations during the seasons were less pronounced.

### Seasonal metagenomic analysis of the 16S rRNA gene

**Figure 7:**
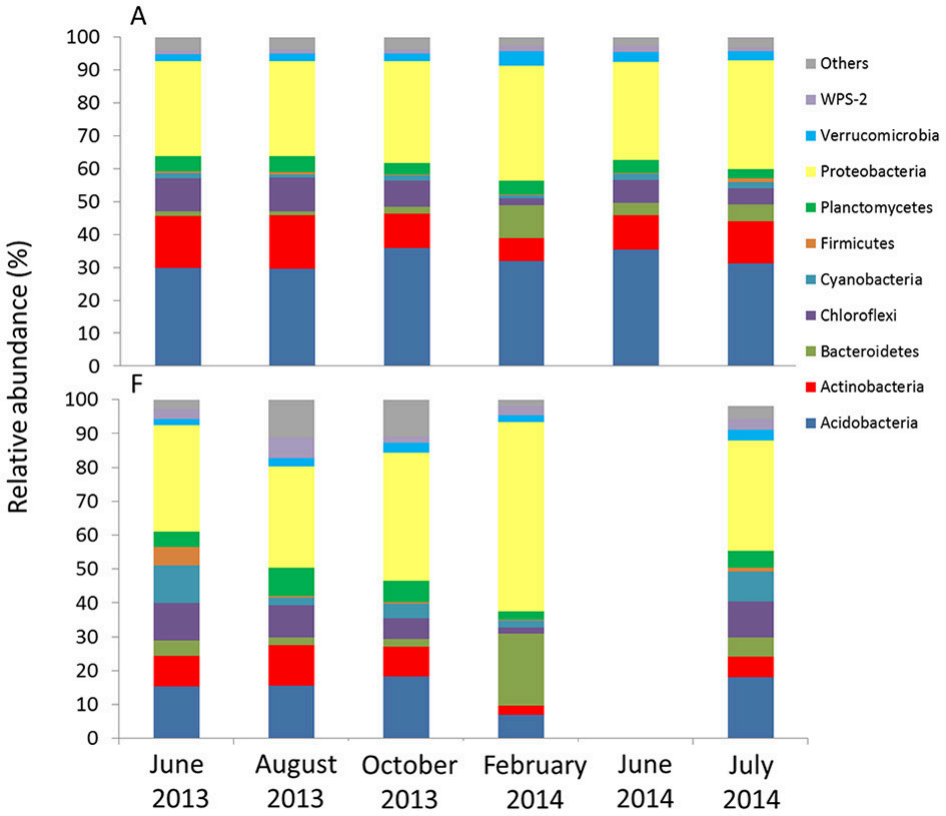
**Relative abundance at the phylum level of phylotypes obtained with high-throughput sequencing for the two sites**. For F, results from June 2014 are not available.

The metagenomic 16S libraries returned 2′727′949 raw sequences. After trimming and sequence cleanup (see Table S3) we recovered a total of 2′160′9101 reads of 464 bp, from which we could assign, through OTU clustering at 97% similarity level, 5881 different 16S rRNA gene phylotypes. The 10 most common groups detected in all the samples belonged to the Phyla Acidobacteria Actinobacteria, Bacteroidetes, Chloroflexi, Cyanobacteria, Firmicutes, Planctomycetes, Proteobacteria, Verrucomicrobia, and WPS-2 (Figure 7, Table S4). The groups with the highest relative abundances at both sites were Acidobacteria (6.8–34.5%) and Proteobacteria (28.7–56.0%). Generally, the other groups remained below 10%. Acidobacteria (Order Solibacterales and Koribacteraceae) had a higher relative abundance (approximating 30%) at A compared to F. Cyanobacterial-related reads were more abundant at F than at A. It also appeared that some phylotypes responded significantly to seasonal change at both sites. For example, *Bacteroidetes*-related reads increased in the February samples. In contrast, *Chloroflexi* experienced a reduction in relative abundance in the same month.

## Discussion

### Effects of seasons and elevation on soil properties

In alpine soils, seasonal variations are expressed by temporal changes in soil properties (Bardgett et al., 2005). For example, climatic variables such as precipitation and snowmelt drive leaching and the loss of important nutrients such as ${\text{NO}}_{3}^{-}$ (Fitzhug et al., 2003). Moreover, organic C has been reported to change seasonally in amount and composition (Wuest, 2014). We observed a general increase in nutrient (e.g., ${\text{NH}}_{4}^{+}$ and DOC) concentrations in October, which may be related to plant senescence and the release of labile compounds to the soil (Bardgett et al., 2005; Jeffries et al., 2009). Such compounds can be utilized by winter communities that stay active under the winter snow cover (Lipson et al., 2000). We also noticed a strong decrease in all of the soluble ions measured below the winter snowpack, suggesting an uptake by the heterotrophic winter communities (Schmidt and Lipson, 2004). However, we did not observe the expected ionic pulse at snowmelt (Lipson et al., 1999; Schmidt and Lipson, 2004; Robson et al., 2007), when the soluble ions stored in the winter snowpack are rapidly flushed into the soil along with the meltwater (Larose et al., 2013; Lazzaro et al., 2015). It has indeed been estimated that 80% of the ions are depleted with the first 30% of meltwater (Larose et al., 2013). Our results suggest that ionic pulses may be extremely fast (days) at our sampling sites, and that most of the soluble compounds had been already taken up by biomass or flushed deeper in the soils by the time we took the samples.

Soil properties also varied significantly across our study sites. We sampled two main ecological domains along a glacier chronosequence, representing a gradient of soil development within the same geological context (Walker et al., 2010). The sites located at the lower elevations (B, T, and A), were strongly vegetated (Table 1); the dominant species (e.g., *A. vulgaris* agg.) reflected location, nutrient status and the different grazing regimes. In contrast, site F exhibited characteristics of a typical pioneer ecosystem in the early stages of soil development, such as oligotrophy and poor soil texture (Lazzaro et al., 2009; Bernasconi et al., 2011).

### Effect of seasons and elevation on gene abundances and on microbial community structure

In our study, seasonal variations appeared to play a more important role in influencing bacterial and fungal communities than site variations, which is in agreement with previous studies at sites with similar seasonal characteristics (Björk et al., 2008; Shahnavaz et al., 2012).

Bacterial gene copy numbers, for example, declined drastically in the months of October and February, and increased slightly from snowmelt to summer. Conversely, fungal copy numbers were highest in February at site F. Such changes suggest that bacterial and fungal communities respond differently to seasonal variations, as shown in previous studies. For example, seasonal changes affected parameters such as microbial biomass in alpine and arctic ecosystems (Lipson et al., 2000; Shahnavaz and Geremia, 2012; Buckeridge et al., 2013). Microbial biomass usually increased in autumn and decreased in spring, depending on C availability (Lipson et al., 1999; Buckeridge et al., 2013). Fungi accounted for most of the winter biomass in tundra soils (Lipson et al., 2002; Schadt et al., 2003). The ability of fungi to decompose phenolic compounds and recalcitrant organic matter (Boer et al., 2005) has been recognized as the main driver of fungal growth in winter soils.

Bacterial community structures at all of our sites tended to shift in parallel with the seasons, with the same dynamics at all locations (Figure 3). At both A and F, we observed a loss of Chloroflexi and Actinobacteria phylotypes in the winter soil samples, whereas Bacteroidetes and Burkholderiales (class β-Proteobacteria) increased. The high relative abundance of copiotrophs under the winter snow suggests high metabolic versatility and adaptation to changing substrates, as noted also by Shahnavaz et al. (2012). The T-RFLP profiles confirmed these trends and indicated that the winter bacterial community is replaced following the spring thaw by a different community. In contrast, fungal communities displayed a different response to seasonal change, as more OTUs were detected in autumn. However, the observed changes in plant and soil nutrient status may trigger these community shifts and gene copy number increases.

As our sampling locations are situated on an ecological gradient characterized by a combination of elevation, topography (A is located in a depression, T on a ridge), plant cover and soil development (i.e., C content), we expected to observe major differences also in microbial community gene copy numbers and structures. Gene copy numbers were one order of magnitude lower at site F, suggesting a correlation with the soil developmental stage. At B, T and A, however, gene copy numbers were similar, and did not show any correlation with plant biomass, species or diversity at the sites. We found no evidence that diversity of plants has an impact on abundance and diversity of microbial communities, as reported elsewhere (Grayston et al., 2001; Wallenstein et al., 2007), not even at our site T which was characterized by a monoculture of *A. vulgaris* agg.

Soil development along glacier chronosequences has been correlated with microbial biomass estimates, at sites ranging from unvegetated soils to alpine meadows (Lazzaro et al., 2009; Zumsteg et al., 2012). Generally, significant increases in microbial biomass are reported with increasing distances from the glacier forefields and along vegetation gradients (e.g., from bare soils to forests; Hahn and Quideau, 2013; Sun et al., 2013).

T-RFLP profiles also failed to reveal community structure shifts along the ecological gradient, as the dominant OTUs detected were the same in all the samples. However, high throughput sequencing revealed some differences in the presence of less dominant phyla. For example, Cyanobacteria (Streptophyta) and Clostridiales, which were found in the F samples, and Acidobacteria, which were found in the samples from A, are commonly found in alpine areas (Mannistö et al., 2006; Zumsteg et al., 2012) and have been recognized as indicator species from Rime et al. (2015). These results indicate out that, along with general heterotrophy, also autotrophic communities of Cyanobacteria can occur during the early stages of soil development. The importance of Cyanobacteria at pioneer sites has been reported by Nemergut et al. (2007).

High-throughput sequencing techniques permit a more detailed insight into community changes than traditional profiling approaches. At various glacier chronosequences, the taxonomic composition of the communities changed according to soil age, with dominant bacterial groups such as Proteobacteria (i.e., alpha and Beta-Proteobacteria) occurring ubiquitously at different soil ages (Philippot et al., 2011; Zumsteg et al., 2012; Rime et al., 2015). Unique phylotypes within the same ubiquitous divisions often characterized different ecological gradients (Lipson, 2006; Rime et al., 2015).

In accordance with the results of this study, however, no significant trends in bacterial community structures were found along glacier chronosequences in the Alps (Lazzaro et al., 2009; Zumsteg et al., 2012), in Canada (Hahn and Quideau, 2013), nor in polar regions (Schütte et al., 2009; Bajerski and Wagner, 2013). Moreover, recent studies indicate that the adaptation mechanisms of bacterial communities to elevational gradients do not necessarily parallel those of plants (Bryant et al., 2008; Fierer et al., 2011). In this context, then, elevation must be considered an indirect proxy for other variable environmental factors that affect bacterial communities.

### Effects of pH on microbial communities

Soil pH is an important soil parameter that is strongly related to nutrient retention in soils and that affects the structures and distribution of microbial communities. The influence of pH on microbial communities has been recognized in a wide variety of soil types (Bryant et al., 2008; Lauber et al., 2009; Rousk et al., 2010; Zinger et al., 2011; Shahnavaz and Geremia, 2012). Such relationships, however, are only clear at a narrow taxonomic scale (i.e., within individual phyla) and along a broad range of soil pH (Lauber et al., 2009).

In our samples, soil pH varied seasonally and at a small spatial scale, especially at the vegetated sites, where it occasionally reached values as low as 3.8. Such an acidic pH reflects the presence of plants and is in agreement with values measured in a neighboring and geologically comparable glacier chronosequence (Bernasconi et al., 2011). Although we did not find any clear relationship between site pH and other soil related parameters, we did find that pH is significantly related to changes in microbial communities particularly in February and at snowmelt. Due to the complex array of environmental variables present at our sampling sites, however, pH could not be considered the only driving environmental factor influencing microbial community structures.

### Seasonal variations and the seasonal snowpack

We observed a seasonal cycle of winter, snowmelt, summer, and autumn at all sites. Seasonal variations did not differ drastically between the sites, despite their different elevations and topographies (Spehn and Körner, 2005). However, we did observe some variability in the thickness and duration of the snowpack at the different sites, as snow tends to melt earlier at the lower elevations. Due to its low thermal conductivity (0.10–0.5 W mK^−1^; Zhang, 2005), snow serves as an efficient insulator protecting the soil beneath the snow from freezing. Soil-air temperature decoupling takes place at 0.4–0.5 m or more of snow cover (Sokratov and Barry, 2002). At one of our sampling sites (A), we verified that, the snow temperature remained at constant values of 0.5°C approximately 60 cm above the soil surface (Figure S3). The soil below (−5cm) displayed temperatures >0°C and therefore did not freeze.

## Conclusion

Alpine regions are characterized by a complex topography, climatic extremes, and pronounced seasons. The temporal and spatial changes of the bacterial and fungal community structures can be adequately monitored by standard molecular methods (T-RFLP, qPCR, and sequencing). In this study we demonstrated that season had a strong effect on microbial community structures and soil properties. At every elevation analyzed, bacterial and fungal community structures exhibited a pronounced annual cycle. Fungal gene copy numbers kept constant throughout the seasons, while bacterial gene copy numbers declined drastically in winter. In contrast to seasons, elevation and vegetation had a minor impact on microbial community structures.

### Conflict of interest statement

The authors declare that the research was conducted in the absence of any commercial or financial relationships that could be construed as a potential conflict of interest.

## Supplementary material

Figure S1 **Temperature and snowpack height from Sept 2012 to Aug 2014, visualizing interannual climatic variations**. For example, winter 2012 (Jan–March) was colder (average air temperature: −6.8°C, snowpack height: 225 cm) than winter 2013 (average air temperature: −3.2°C, snowpack height: 162 cm).

Figure S2 **Time lag analysis on the basis of pairwise Bray-Curtis dissimilarities of the T-RFLP profiles of the bacterial 16S and fungal 18S rRNA genes**.

Figure S3 **Air, snowpack, and soil temperatures at site A, from 25 Jan to 6, Feb 2014**. Measurements were taken over 6 days using Ibuttons® (Measurement Systems Ltd, Newbury, UK) temperature loggers installed on a wooden stick placed at different depths of the snowpack and soil. During the measurement period, the snowpack height was approximately 155 cm.

Table S1 **Sampling dates at each site**.

Table S2 **Primers used in this study for each methodology**.

Table S3 **Pipeline of metagenomic analysis (developed by the Genetic Diversity Center GDC, ETH Zurich)**.

Table S4 **Relative abundances of dominant phylotypes found through Illumina high throughput sequencing of the 16S rRNA gene from A and F**. Shading indicates the seasons and samples where a certain phylotype was most abundant.
