# Supplementary figures and images for: Structures of Microbial Communities in Alpine Soils: Seasonal and Elevational Effects
Source: Front Microbiol. 2015 Nov 26;6:1330. doi: 10.3389/fmicb.2015.01330 (PMC4660872; doi:10.3389/fmicb.2015.01330)

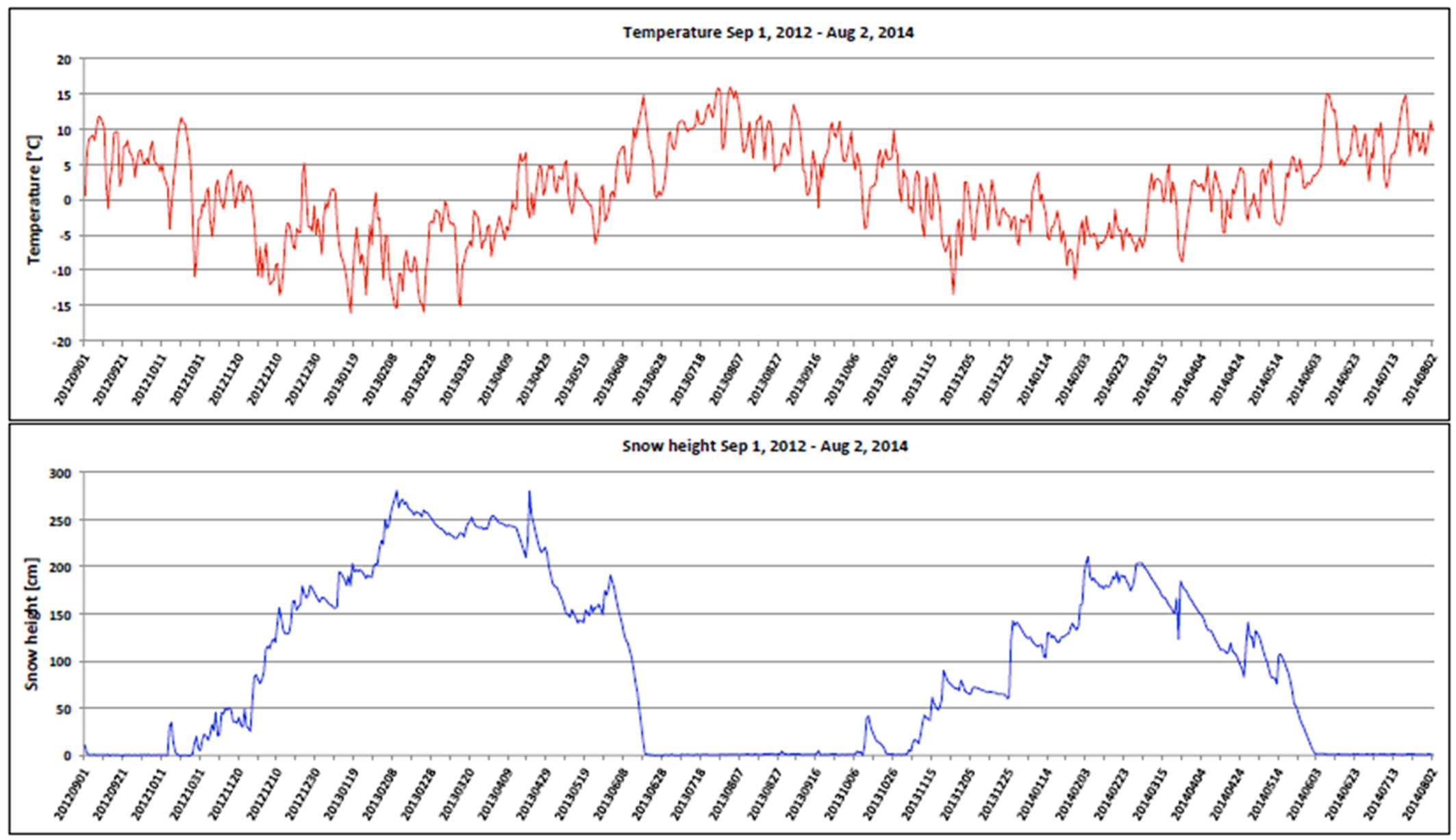

Supplement: Figure S1 — Temperature and snowpack height from Sept 2012 to Aug 2014, visualizing interannual climatic variations. For example, winter 2012 (Jan–March) was colder (average air temperature: −6.8°C, snowpack height: 225 cm) than winter 2013 (average air temperature: −3.2°C, snowpack height: 162 cm). [file Image1.TIFF]

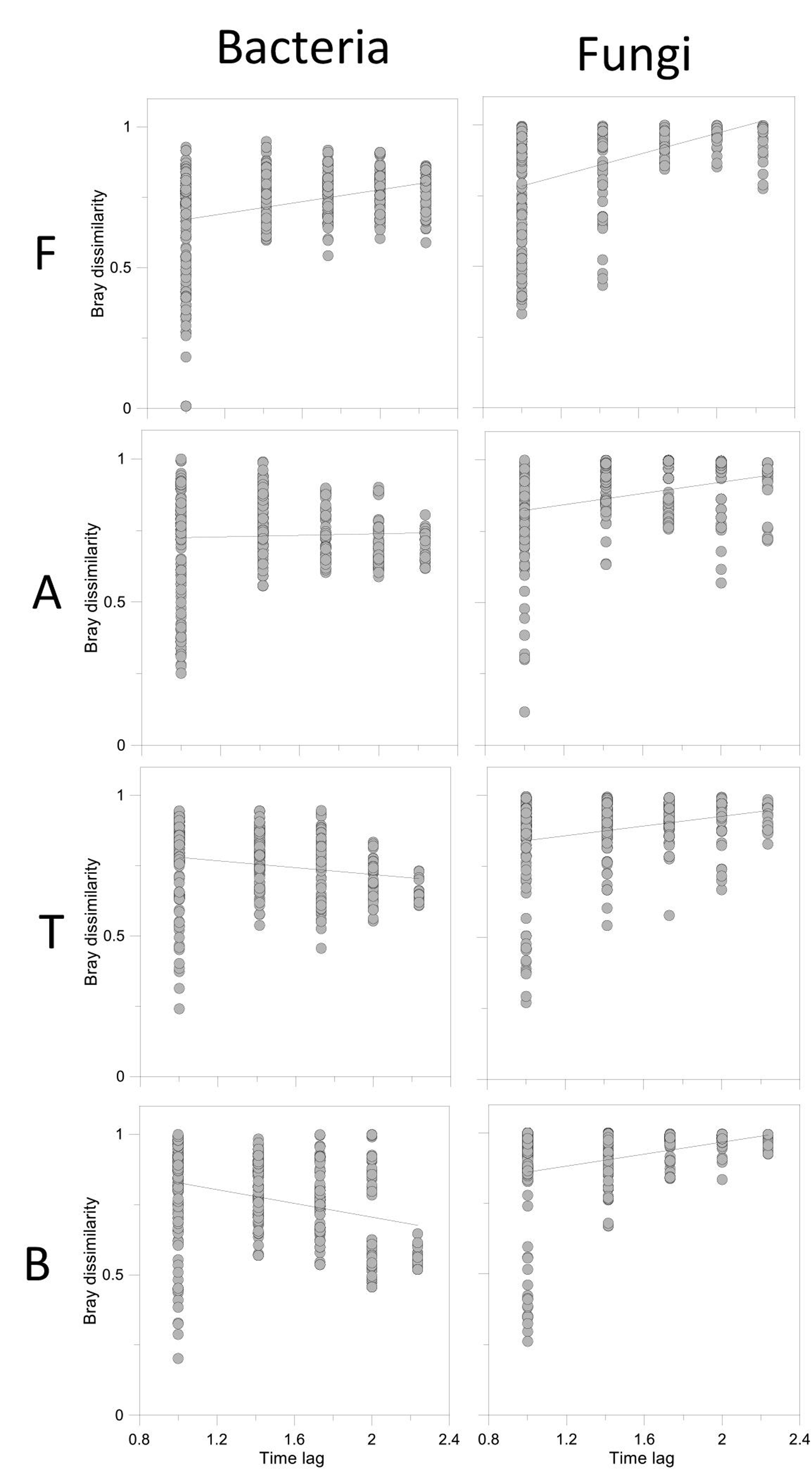

Supplement: Figure S2 — Time lag analysis on the basis of pairwise Bray-Curtis dissimilarities of the T-RFLP profiles of the bacterial 16S and fungal 18S rRNA genes. [file Image2.TIFF]

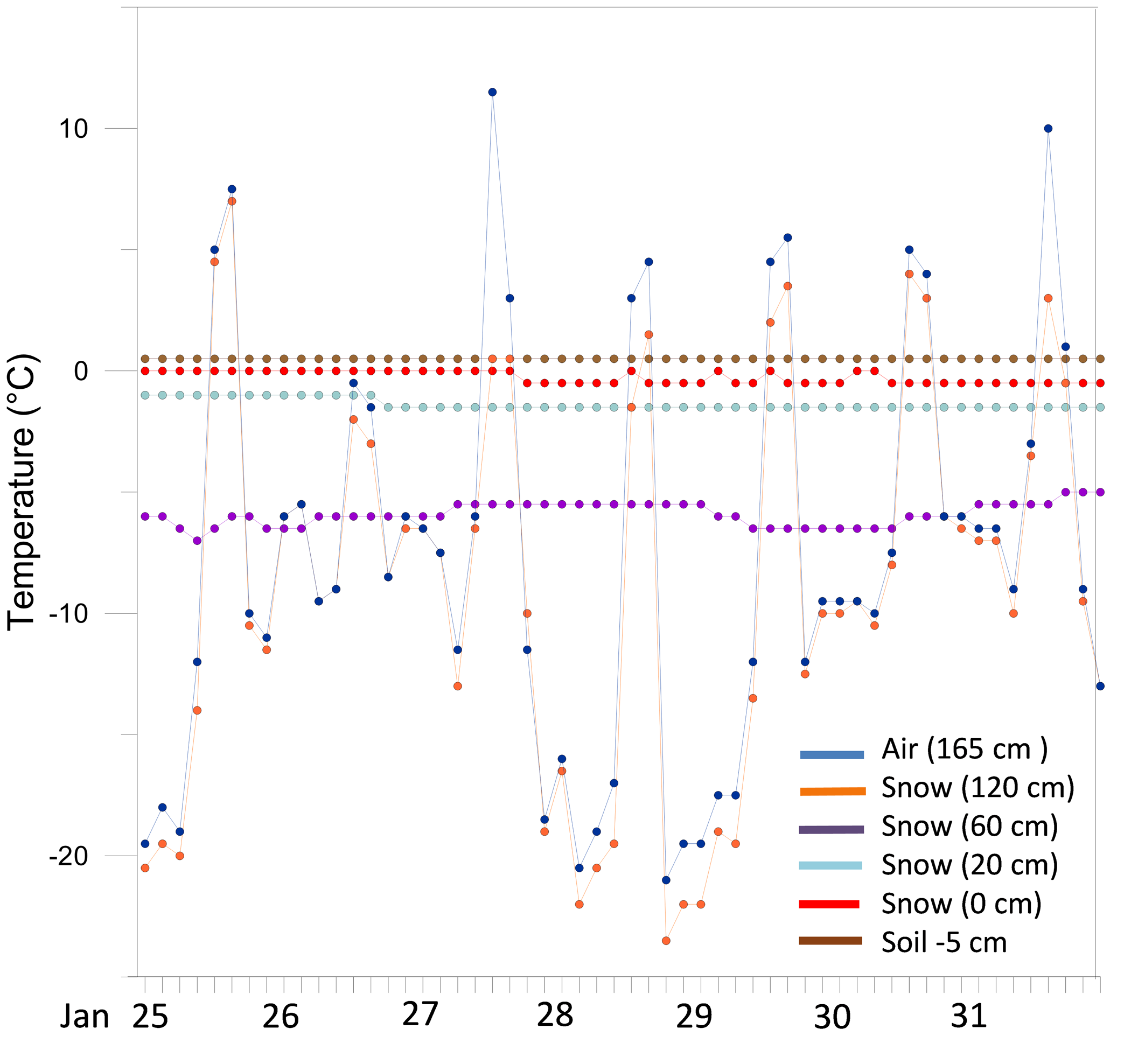

Supplement: Figure S3 — Air, snowpack, and soil temperatures at site A, from 25 Jan to 6, Feb 2014. Measurements were taken over 6 days using Ibuttons® (Measurement Systems Ltd, Newbury, UK) temperature loggers installed on a wooden stick placed at different depths of the snowpack and soil. During the measurement period, the snowpack height was approximately 155 cm. [file Image3.TIF]
