# Supplementary material for: Structures of Microbial Communities in Alpine Soils: Seasonal and Elevational Effects
Source: Front Microbiol. 2015 Nov 26;6:1330. doi: 10.3389/fmicb.2015.01330 (PMC4660872; doi:10.3389/fmicb.2015.01330)
Supplement: Table S1 — Sampling dates at each site. [file Table1.DOCX]

***Supplementary Material***

**Structures of microbial communities in alpine soils: seasonal and elevational effects**

**Anna Lazzaro*, Daniela Hilfiker, Josef Zeyer**

Environmental Microbiology, Institute of Biogeochemistry and Pollutant Dynamics, ETH Zurich, Universitätstrasse 16, 8092 Zurich

*Corresponding author:

Anna Lazzaro

Environmental Microbiology

Institute of Biogeochemistry and Pollutant Dynamics

ETH Zurich

Universitätstrasse 16

8092 Zurich, Switzerland

Email: anna.lazzaro@env.ethz.ch

Tel: +41446336045

**1. Supplementary Tables**

**Table S1.** Sampling dates at each site

| **Site** | **2013** | | |  | **2014** | | |
| --- | --- | --- | --- | --- | --- | --- | --- |
|  | **Spring snowmelt** | **Summer snow-free** | **Autumn snow-free** |  | **Winter snow-covered** | **Spring snowmelt** | **Summer snow-free** |
| F | 18^th^ Jun | 23^th^ Aug | 25^th^ Oct |  | 12^th^ Feb | 1^st^ Jun | 27^st^ Jul |
| A | 12^th^ Jun | 23^th^ Aug | 25^th^ Oct |  | 12^th^ Feb | 1^st^ Jun | 27^st^ Jul |
| T | 12^th^ Jun | 23^th^ Aug | 25^th^ Oct |  | 3^d^ Feb | 1^st^ Jun^1^ | 27^st^ Jul |
| B | 12^th^ Jun | 23^th^ Aug | 25^th^ Oct |  | 3^d^ Feb | 31^st^ May^1^ | 27^st^ Jul |

^1^at B and T, snow had just melted one day before
