# Supplementary material for: Structures of Microbial Communities in Alpine Soils: Seasonal and Elevational Effects
Source: Front Microbiol. 2015 Nov 26;6:1330. doi: 10.3389/fmicb.2015.01330 (PMC4660872; doi:10.3389/fmicb.2015.01330)
Supplement: Table S2 — Primers used in this study for each methodology. [file Table2.DOCX]

***Supplementary Material***

**Structures of microbial communities in alpine soils: seasonal and elevational effects**

**Anna Lazzaro*, Daniela Hilfiker, Josef Zeyer**

Environmental Microbiology, Institute of Biogeochemistry and Pollutant Dynamics, ETH Zurich, Universitätstrasse 16, 8092 Zurich

*Corresponding author:

Anna Lazzaro

Environmental Microbiology

Institute of Biogeochemistry and Pollutant Dynamics

ETH Zurich

Universitätstrasse 16

8092 Zurich, Switzerland

Email: anna.lazzaro@env.ethz.ch

Tel: +41446336045

**Table S2.** Primers used in this study for each methodology

| **Method** | **Primer name** | **Target** | **Sequence** | **Reference** |
| --- | --- | --- | --- | --- |
| T-RFLP | 27F | Bacterial 16S rRNA gene | 5’-AGAGTTTGATCMTGGCTCAG-3’ | Winsley et al., 2012 |
|  | 1064 Rev |  | 5’-AYCTCACGRCACGAGCTGAC-3’ | Winsley et al., 2012 |
|  | nu-SSU-0817 | Fungal 18S rRNA gene | TTAGCATGGAATAATRRAATAGGA | Borneman and Hartin, 2000 |
|  | FR1 |  | 5′-AICCATTCAATCGGTAIT-3′ | Chemidlin Prévost-Bouré et al., 2011 |
| qPCR | 349 F | Bacterial 16S rRNA gene | 5’-AGGCAGCAGTDRGGAAT-3’ | Takai and Horikoshi, 2000 |
|  | 806 Rev |  | 5’-GGACTACYVGGGTATCTAAT-3’ | Takai and Horikoshi, 2000 |
|  | FR1 | Fungal 18S rRNA gene | 5′-AICCATTCAATCGGTAIT-3′ | Chemidlin Prévost-Bouré et al., 2011). |
|  | FF390 |  | 5′-CGATAACGAACGAGACCT-3′ | Chemidlin Prévost-Bouré et al., 2011). |
| Illumina amplicon sequencing | Bakt_341F | Bacterial 16S rRNA gene | 5'-CCTACGGGNGGCWGCAG-3' | Herlemann et al. 2011 |
|  | Bakt_805R |  | 5'-GACTACHVGGGTATCTAATCC-3' | Herlemann et al. 2011 |
