# Supplementary material for: Structures of Microbial Communities in Alpine Soils: Seasonal and Elevational Effects
Source: Front Microbiol. 2015 Nov 26;6:1330. doi: 10.3389/fmicb.2015.01330 (PMC4660872; doi:10.3389/fmicb.2015.01330)
Supplement: Table S3 — Pipeline of metagenomic analysis (developed by the Genetic Diversity Center GDC, ETH Zurich). [file Table3.DOCX]

***Supplementary Material***

**Structures of microbial communities in alpine soils: seasonal and elevational effects**

**Anna Lazzaro*, Daniela Hilfiker, Josef Zeyer**

Environmental Microbiology, Institute of Biogeochemistry and Pollutant Dynamics, ETH Zurich, Universitätstrasse 16, 8092 Zurich

*Corresponding author:

Anna Lazzaro

Environmental Microbiology

Institute of Biogeochemistry and Pollutant Dynamics

ETH Zurich

Universitätstrasse 16

8092 Zurich, Switzerland

Email: anna.lazzaro@env.ethz.ch

Tel: +41446336045

**Table S3**

Pipeline of metagenomic analysis (developed by the Genetic Diversity Center GDC, ETH Zurich)

| **Step** | **Process** | **Software** | **Reference** |
| --- | --- | --- | --- |
| 1 | quality control | FastQC v0.10.1 | http: // www.bioinformatics.bbsrc.ac.uk/ projects/ fastqc/ |
| 2 | merging | FLASH v1.4.2 |  |
| 3 | primer trimming | cutadapt v1.4.2 | (DOI: http://dx.doi.org/10.14806/ej.17.1.200) |
| 4 | phylotype clustering | Usearch v7.0. 1090_i86linux64 | Edgar (2010)^1^ |
| 5 | BLAST and taxonomic assignment | Greengenes database v13_5. |  |

^1^References: Edgar RC. Search and clustering orders of magnitude faster than BLAST. Bioinformatics 2010; 26: 2460–2461.
