# Supplementary material for: Structures of Microbial Communities in Alpine Soils: Seasonal and Elevational Effects
Source: Front Microbiol. 2015 Nov 26;6:1330. doi: 10.3389/fmicb.2015.01330 (PMC4660872; doi:10.3389/fmicb.2015.01330)
Supplement: Table S4 — Relative abundances of dominant phylotypes found through Illumina high throughput sequencing of the 16S rRNA gene from A and F. Shading indicates the seasons and samples where a certain phylotype was most abundant. [file Table4.DOCX]

***Supplementary Material***

**Structures of microbial communities in alpine soils: seasonal and elevational effects**

**Anna Lazzaro*, Daniela Hilfiker, Josef Zeyer**

Environmental Microbiology, Institute of Biogeochemistry and Pollutant Dynamics, ETH Zurich, Universitätstrasse 16, 8092 Zurich

*Corresponding author:

Anna Lazzaro

Environmental Microbiology

Institute of Biogeochemistry and Pollutant Dynamics

ETH Zurich

Universitätstrasse 16

8092 Zurich, Switzerland

Email: anna.lazzaro@env.ethz.ch

Tel: +41446336045

**Table S4.** Relative abundances of dominant phylotypes found through Illumina high throughput sequencing of the 16S rRNA gene from A and F. Shading indicates the seasons and samples where a certain phylotype was most abundant.

| **Sampling month** | **June-2013** | **June-2013** | **Aug-2013** | **Aug-2013** | **Oct-2013** | **Oct-2013** | **Feb-2014** | **Feb-2014** | **June-2014** | **July-2014** | **July-2014** |
| --- | --- | --- | --- | --- | --- | --- | --- | --- | --- | --- | --- |
| **Site** | **A** | **F** | **A** | **F** | **A** | **F** | **A** | **F** | **A** | **A** | **F** |
|  |  |  |  |  |  |  |  |  |  |  |  |
| **p__Acidobacteria** | 28.42 | 14.52 | 28.33 | 13.92 | 34.49 | 15.65 | 31.46 | 6.81 | **33.44** | 29.23 | 16.43 |
| c__Acidobacteriia | 14.84 | 7.76 | 14.36 | 5.70 | 16.34 | 5.29 | 19.22 | 4.97 | **18.10** | 15.67 | 7.27 |
| o__Acidobacteriales | 14.84 | 7.76 | 14.36 | 5.70 | 16.34 | 5.29 | 19.22 | 4.97 | **18.10** | 15.67 | 7.27 |
| c__DA052 | 5.89 | 0.87 | 6.28 | 1.33 | **9.61** | 1.76 | 5.48 | 0.14 | 6.52 | 5.57 | 1.28 |
| o__Ellin6513 | 5.89 | 0.87 | 6.28 | 1.33 | **9.61** | 1.76 | 5.48 | 0.14 | 6.52 | 5.57 | 1.28 |
| c__iii1-8 | 1.34 | 0.36 | 1.35 | **1.51** | 0.73 | 2.42 | 0.21 | 0.09 | 0.58 | 0.71 | 1.25 |
| o__32-20 | 0.72 | 0.20 | 0.81 | 0.41 | 0.24 | **0.59** | 0.12 | 0.04 | 0.28 | 0.38 | 0.27 |
| o__DS-18 | 0.15 | 0.14 | 0.15 | **0.95** | 0.19 | 1.51 | 0.02 | 0.04 | 0.08 | 0.16 | 0.90 |
| c__Solibacteres | 6.35 | 5.53 | 6.34 | 5.38 | 7.82 | 6.19 | 6.55 | 1.61 | **8.24** | 7.28 | 6.62 |
| o__Solibacterales | 6.31 | 5.50 | 6.30 | 5.32 | 7.72 | 6.08 | 6.53 | 1.61 | **8.18** | 7.24 | 6.54 |
| **p__Actinobacteria** | 15.91 | 9.20 | **16.12** | 11.83 | 10.41 | 8.68 | 7.07 | 2.62 | 10.48 | 12.97 | 6.11 |
| c__Acidimicrobiia | 3.12 | 1.25 | **3.35** | 3.74 | 2.67 | 2.85 | 1.32 | 0.33 | 2.24 | 2.30 | 1.97 |
| o__Acidimicrobiales | 3.12 | 1.25 | 3.35 | **3.74** | 2.67 | 2.85 | 1.32 | 0.33 | 2.24 | 2.30 | 1.97 |
| c__Actinobacteria | **9.87** | 6.06 | 9.65 | 3.33 | 6.47 | 2.88 | 4.90 | 1.82 | 6.72 | 7.97 | 2.76 |
| o__Actinomycetales | **9.87** | 6.06 | 9.65 | 3.32 | 6.47 | 2.88 | 4.90 | 1.81 | 6.71 | 7.97 | 2.75 |
| c__Thermoleophilia | 2.91 | 1.89 | 3.12 | **4.77** | 1.28 | 2.95 | 0.85 | 0.46 | 1.53 | 2.70 | 1.38 |
| o__Gaiellales | 0.42 | 0.51 | 0.43 | **2.44** | 0.16 | 1.98 | 0.11 | 0.10 | 0.16 | 0.89 | 0.65 |
| o__Solirubrobacterales | 2.49 | 1.38 | **2.69** | 2.33 | 1.11 | 0.97 | 0.74 | 0.36 | 1.37 | 1.82 | 0.72 |
| **p__Chloroflexi** | 8.67 | **8.95** | 8.67 | 3.37 | 6.76 | 1.03 | 1.36 | 1.30 | 6.07 | 3.67 | 7.64 |
| c__Ktedonobacteria | 8.67 | **8.95** | 8.67 | 3.37 | 6.76 | 1.03 | 1.36 | 1.30 | 6.07 | 3.67 | 7.64 |
| o__B12-WMSP1 | 0.04 | 0.11 | 0.04 | **0.54** | 0.03 | 0.10 | 0.02 | 0.02 | 0.01 | 0.01 | 0.12 |
| o__JG30-KF-AS9 | 0.25 | 0.01 | 0.24 | 0.02 | **0.67** | 0.02 | 0.09 | 0.00 | 0.46 | 0.37 | 0.03 |
| o__Ktedonobacterales | 2.60 | **6.24** | 2.69 | 2.06 | 2.13 | 0.57 | 0.53 | 0.68 | 1.92 | 1.31 | 4.74 |
| o__Thermogemmatisporales | **5.76** | 2.59 | 5.69 | 0.75 | 3.90 | 0.34 | 0.71 | 0.60 | 3.66 | 1.95 | 2.76 |
| **p__Cyanobacteria** | 1.27 | **10.28** | 0.76 | 1.74 | 0.99 | 4.14 | 0.36 | 1.12 | 1.19 | 1.50 | 8.13 |
| c__Chloroplast | 1.27 | **10.28** | 0.76 | 1.74 | 0.99 | 4.14 | 0.36 | 1.12 | 1.19 | 1.50 | 8.13 |
| o__Chlorophyta | 0.01 | **1.17** | 0.01 | 0.19 | 0.03 | 0.24 | 0.05 | 0.32 | 0.10 | 0.04 | 0.69 |
| o__Streptophyta | 1.25 | **8.98** | 0.75 | 1.51 | 0.95 | 3.85 | 0.26 | 0.75 | 1.04 | 1.43 | 7.34 |
| **p__Planctomycetes** | 4.16 | 3.49 | 4.45 | **6.91** | 2.69 | 5.14 | 2.50 | 1.13 | 3.17 | 2.18 | 3.13 |
| c__Planctomycetia | 4.16 | 3.49 | 4.45 | **6.91** | 2.69 | 5.14 | 2.50 | 1.13 | 3.17 | 2.18 | 3.13 |
| o__Gemmatales | 3.66 | 3.13 | 3.97 | **5.57** | 2.36 | 3.99 | 2.04 | 1.04 | 2.75 | 1.80 | 2.48 |
| o__Pirellulales | 0.34 | 0.13 | 0.32 | **0.52** | 0.19 | 0.48 | 0.13 | 0.03 | 0.23 | 0.26 | 0.23 |
| o__Planctomycetales | 0.16 | 0.23 | 0.16 | **0.82** | 0.14 | 0.67 | 0.33 | 0.05 | 0.19 | 0.12 | 0.42 |
| **p__Proteobacteria** | 28.77 | 31.51 | 28.93 | 29.84 | 30.95 | 37.56 | 34.90 | **56.01** | 29.72 | 32.90 | 32.62 |
| c__Alphaproteobacteria | 17.26 | 15.36 | **17.43** | 15.88 | 15.14 | 15.06 | 14.41 | 13.37 | 14.55 | 15.48 | 13.39 |
| o__ | 0.24 | 0.17 | 0.21 | 0.44 | 0.32 | 0.44 | **0.79** | 0.40 | 0.26 | 0.28 | 0.34 |
| o__Caulobacterales | 0.47 | 1.40 | 0.41 | 0.69 | 0.84 | 0.56 | 1.93 | **2.24** | 1.15 | 1.06 | 1.55 |
| o__Ellin329 | 0.37 | 0.66 | 0.41 | 1.18 | 0.35 | 1.47 | **1.68** | 0.60 | 0.50 | 0.51 | 1.45 |
| o__Rhizobiales | 11.80 | 5.51 | **12.16** | 8.18 | 8.54 | 7.18 | 4.59 | 2.85 | 8.38 | 8.89 | 5.24 |
| o__Rhodospirillales | 3.94 | **5.38** | 3.90 | 3.91 | 4.56 | 4.04 | 4.74 | 2.81 | 3.86 | 3.87 | 3.19 |
| o__Rickettsiales | 0.33 | 0.94 | 0.25 | 0.45 | 0.39 | 0.64 | 0.23 | 0.14 | 0.30 | 0.56 | **0.65** |
| o__Sphingomonadales | 0.09 | 1.28 | 0.06 | 0.97 | 0.12 | 0.66 | 0.41 | **4.31** | 0.09 | 0.23 | 0.96 |
| c__Betaproteobacteria | 3.44 | 7.29 | 3.47 | 4.40 | 6.00 | 8.23 | 5.97 | **25.73** | 5.73 | 6.78 | 4.85 |
| o__ | 0.28 | 0.07 | 0.25 | 0.38 | 0.28 | **0.64** | 0.49 | 0.09 | 0.21 | 0.25 | 0.23 |
| o__A21b | 1.69 | 0.25 | 1.83 | 0.31 | 4.22 | 0.34 | 0.85 | 0.14 | 3.74 | **3.99** | 0.37 |
| o__Burkholderiales | 1.08 | **6.67** | 1.01 | 2.53 | 1.09 | 5.32 | 4.38 | 25.41 | 1.50 | 1.93 | 3.48 |
| o__Ellin6067 | 0.09 | 0.16 | 0.08 | 0.57 | 0.07 | **0.83** | 0.03 | 0.02 | 0.03 | 0.19 | 0.38 |
| o__SC-I-84 | 0.21 | 0.09 | 0.20 | 0.37 | 0.28 | **0.57** | 0.19 | 0.04 | 0.19 | 0.32 | 0.19 |
| c__Deltaproteobacteria | 4.65 | 3.21 | 4.77 | 4.45 | 5.49 | 8.19 | 4.09 | 2.98 | 4.43 | 4.21 | 5.57 |
| o__Bdellovibrionales | 0.13 | 0.17 | 0.11 | 0.28 | 0.20 | 0.20 | **0.52** | 0.30 | 0.17 | 0.17 | 0.32 |
| o__Desulfuromonadales | 0.12 | 0.51 | 0.16 | 0.81 | 0.07 | **4.57** | 0.02 | 0.00 | 0.11 | 0.23 | 0.77 |
| o__Myxococcales | 2.63 | 2.18 | 2.66 | 2.42 | 3.08 | 2.49 | 2.32 | 2.24 | 2.97 | 2.90 | **3.76** |
| o__Syntrophobacterales | 1.53 | 0.08 | 1.61 | 0.35 | **1.89** | 0.46 | 0.65 | 0.01 | 0.93 | 0.71 | 0.17 |
| c__Gammaproteobacteria | 3.42 | 5.66 | 3.26 | 5.12 | 4.31 | 6.09 | 10.43 | 13.94 | 5.02 | 6.43 | 8.81 |
| o__Legionellales | 0.25 | 0.20 | 0.27 | **1.21** | 0.43 | 0.97 | 0.30 | 0.22 | 0.37 | 0.26 | 0.62 |
| o__Pseudomonadales | 0.06 | 1.16 | 0.05 | 0.12 | 0.42 | 1.09 | 2.63 | **9.90** | 0.96 | 1.83 | 2.40 |
| o__Xanthomonadales | 3.11 | 4.28 | 2.94 | 3.76 | 3.40 | **3.99** | 7.45 | 3.64 | 3.68 | 4.33 | 5.79 |
| **p__Verrucomicrobia** | 1.09 | 1.06 | 1.12 | 1.07 | 1.00 | 1.14 | 1.00 | 0.78 | 1.43 | 1.24 | **1.38** |
| c__[Spartobacteria] | 1.09 | 1.06 | 1.12 | 1.07 | 1.00 | 1.14 | 1.00 | 0.78 | 1.43 | 1.24 | **1.38** |
| o__[Chthoniobacterales] | 1.09 | 1.06 | 1.12 | 1.07 | 1.00 | 1.14 | 1.00 | 0.78 | **1.43** | 1.24 | 1.38 |
| **p__WPS-2** | 1.16 | 2.90 | 1.27 | **6.06** | 1.20 | 1.83 | 1.65 | 2.70 | 1.74 | 1.21 | 3.12 |
| c__ | 1.16 | 2.90 | 1.27 | **6.06** | 1.20 | 1.83 | 1.65 | 2.70 | 1.74 | 1.21 | 3.12 |
| o__ | 1.16 | 2.90 | 1.27 | **6.06** | 1.20 | 1.83 | 1.65 | 2.70 | 1.74 | 1.21 | 3.12 |
